# Supplementary material for: Prevalence and health consequences of nonmedical use of tramadol in Africa: A systematic scoping review
Source: PLOS Glob Public Health. 2024 Jan 18;4(1):e0002784. doi: 10.1371/journal.pgph.0002784 (PMC10796000; doi:10.1371/journal.pgph.0002784)
Supplement: S1 Table — (DOCX) [file pgph.0002784.s002.docx]

**S2 Table PICOS search criteria and sources for the review**

| **PICOS** | **Definition** |
| --- | --- |
| Populations | All African populations, regardless of their age, place of residence (rural & urban), and socio-economic status. |
| Interventions | All studies investigating the prevalence of substance use encompassing instances of nonmedical tramadol use, as well as research focusing on the prevalence of tramadol use within distinct African population categories, comprise either qualitative or quantitative studies investigating the health consequences associated with nonmedical tramadol use. |
| Comparator | Health consequences were grouped using the WHO's International Classification of Diseases-11 (ICD-11), and subpopulation categories grouped prevalence. |
| Outcome | Health consequences of NMU of tramadol or prevalence use among subpopulations in Africa |
| Settings | Any settings |
| Study design | Cross-sectional, qualitative, retrospective, prospective, case studies, cohort studies. |
| Date | No time limit |
| Databases | PubMed/Medline, Web of Science, Scopus, African Journal Online Database, Global Health (EBSCO), Google Scholar. |
| Exclusions | 1) Studies without a specific indicator (prevalence, mortality, morbidity, etc.).  2) Interventional and quasi-experimental studies or in vitro studies.  3) Studies in which tramadol is reported in combination with other drugs so that it is impossible to specify the isolated effects of tramadol.  4) Texts and opinion literature.  5) Study with wrong populations (not in Africa)  6) Studies not found |
