# Supplementary material for: Prevalence and health consequences of nonmedical use of tramadol in Africa: A systematic scoping review
Source: PLOS Glob Public Health. 2024 Jan 18;4(1):e0002784. doi: 10.1371/journal.pgph.0002784 (PMC10796000; doi:10.1371/journal.pgph.0002784)
Supplement: S2 Table — (DOCX) [file pgph.0002784.s003.docx]

**S3 Table. Search Strategy in databases**

|  | Search string | Data of Search | Number of results | Selectionned for review |
| --- | --- | --- | --- | --- |
| Medline | (tramadol[Title/Abstract]) AND (Africa) | 19-janv-23 | 108 | 108 |
| Web of Science | ("tramadol") (Topic) AND africa (All Fields) | 06-janv-23 | 72 | 72 |
| Scopus | ( TITLE ( tramadol ) AND TITLE-ABS-KEY ( ( "abuse" OR "non-medical use" OR "abuse" ) ) AND ALL ( africa ) ) | 06-janv-23 | 15 | 15 |
| African Journal Online Database | ("tramadol") AND ("Non-medical use" OR "misuse" OR "abuse*") | 19-janv-23 | 178 | 178 |
| Google Scholar | ("tramadol") AND ("Non-medical use" OR "misuse" OR "abuse*") AND ("Africa") | 20-janv-23 | 3660 | 317 |
| Global Health (EBSCO) | tramadol AND ( misuse or abuse or addiction or overuse ) AND Africa | 29-avr-23 | 32 | 32 |
| Total | 722 | | | |
