# Supplementary material for: Prevalence and health consequences of nonmedical use of tramadol in Africa: A systematic scoping review
Source: PLOS Glob Public Health. 2024 Jan 18;4(1):e0002784. doi: 10.1371/journal.pgph.0002784 (PMC10796000; doi:10.1371/journal.pgph.0002784)
Supplement: S3 Table — (DOCX) [file pgph.0002784.s004.docx]

| **N^0^** | **Authors** | **Country of study** | **Sub-population/Target population concerned** | **Type of Study** | **Health Consequences of NMU of tramadol** | **% (Pop 1)^1^** | **% (Pop 2)^2^** | **% (Pop 3)^3^** |
| --- | --- | --- | --- | --- | --- | --- | --- | --- |
| 1 | Abbas RA et al. (2013) [1] | Egypt | Temporary hired hospital cleaners | Cohort study | Not reported | Temporary cleaners (n=242)  39.7% | Permanent cleaners (n=209)  20.6% |  |
| 2 | Abdel Kareem & Ali, (2018) [2] | Egypt | Drivers (Taxi, microbus, private cars, truck drivers) | Cross-sectional study | Not reported | Drivers (n=3338)  7.2% |  |  |
| 3 | Abdel Moneim et al. (2017) [3] | Egypt | Trauma victims | Cross-sectional study | Not reported | Trauma patient (n=300)  8.3% |  |  |
| 4 | Abdelfattah et al. (2019) [4] | Egypt | Students/ Benha University | Cross-sectional study | Not reported | University Student (16/283)  5.7% |  |  |
| 5 | Abdelhamid et al. (2022) [5] | Egypt | Patients scheduled for elective orthopedic, general, vascular, and plastic surgeries | Cohort study | Not reported | Patients (25/500)  5% |  |  |
| 6 | Abd-Elkader MR et al. (2020) [6] | Egypt | Minibus Drivers, Construction and textile industries workers | Cross-sectional study | Not reported | Construction workers (n=300)  92.3% | Textile industries workers (n=300)  53.0% | Minibus Drivers (n=300)  25.3% |
| 7 | Abd-Elwahab & Amin (2012) [7] | Egypt | Individuals with substance abuses | Cohort study | Not reported | Cases: Individuals with substance abuses  96% |  |  |
| 8 | AbdelWahab et al. (2018) [8] | Egypt | Treatment-Seeking men | Cross-sectional study | The strongest motive for drug continuation (pain avoidance, pleasure-seeking, habit) |  |  |  |
| 9 | Agberotimi et al. (2020) [9] | Nigeria | Skilled workers (Hairstylists and mechanics) | Cross-sectional study | Not reported | Hairstylists (n=64)  10.1% | Mechanics (n=54)  13.3% | Overall (n=118)  23.4% |
| 10 | Amankwaa (2019) [10] | Ghana | Senior high school students | Cross-sectional study | Not reported | Senior high school students (n=418)  31.0% |  |  |
| 11 | Argungu et al. (2021) [11] | Nigeria | Patients with a history of tramadol abuse | Case-control study | Sexual esteem, sexual depression and sexual preoccupation were significantly better in the control group than in the tramadol abuse group. |  |  |  |
| 12 | Azab et al. (2022) [12] | Egypt | Patients presenting acute intoxication (unintentional poisoning) | Retrospective Study | Not reported | Patients presenting acute intoxication (n = 11,281)  37.5% |  |  |
| 13 | Bassiony & Seleem (2020) [13] | Egypt | Patients with substances use disorders | Cross-sectional study | Not reported | Patients with substances use disorders (n= 100)  92% |  |  |
| 14 | Bassiony et al. (2021) [14] | Egypt | Egyptian adolescents with opioid use disorders attributed to Tramadol (OUD-T) | Cross-sectional study | Approximately 38% of adolescents with tramadol misuse had ADHD (attention-deficit/hyperactivity disorder); 50% had hyperactive type ADHD, while 24% and 26% had inattentive and combined type ADHD, respectively. |  |  |  |
| 15 | Bassiony et al. (2015) [15] | Egypt | School Student (public) | Cross-sectional study | Not reported | Public School Student (n=204)  8.8% |  |  |
| 16 | Bassiony MM et al. (2016) [16] | Egypt | Patient with OUD-T | Case-control study | 49% had comorbid psychiatric disorders, 59.2% had mood, 38.8 had anxiety, and 26.5% had psychotic disorders.  Mood disorders include Major depression (8%), bipolar disorder (3%), dysthymia (2%) and substance-induced mood disorders (19%)  Anxiety disorders included specific phobia (5%), social phobia (3%), dysthymia (2%), posttraumatic stress disorders (1%), obsessive-compulsive disorder (1%), generalized anxiety disorder (1%), and agoraphobia (1%). |  |  |  |
| 17 | Bassiony MM et al. (2022) [17] | Egypt | Adults with opioid use disorders attributed to Tramadol | Cross-sectional study | Thirty‐eight cases (31%) had adult attention-deficit/Hyperactivity disorder (ADHD), and most had inattentive or mixed types. Long and high use dose of Tramadol is associated with ADHD. |  |  |  |
| 18 | Bassiony, Abdelghani, et al. (2018) [18] | Egypt | University Students | Cross-sectional study | Not reported | All students (n=1173)  12.3% | Students with substance use (n=264)  21% |  |
| 19 | Bassiony, Salah El-Deen, et al. (2018) [19] | Egypt | University Students | Cross-sectional study | Not reported | All University students (n=1176)  12.3% | All students who used substances (n= 265)  54.7% |  |
| 20 | Bio-Sya A et al. (2022) [20] | Benin | Secondary School Students | Cross-sectional study | Not reported | A student with a history of substance abuse (n=221)  1.81% |  |  |
| 21 | Bio-Sya et al. (2021) [21] | Benin | Secondary school students | Cross-sectional study | Not reported | Secondary school students (Public & Private) (n=384)  9.6% (13.4% for males & 4.4% for females) |  |  |
| 22 | Burbwa & Kimbi (2019) [22] | Nigeria | Riders business youth | Cross-sectional study | Not reported | Riders business (n=214)  56% |  |  |
| 23 | Carmel et al. (2019) [23] | Togo | Motorcycle taxi drivers | Cross-sectional study | Convulsions rise (33.3%), headaches (60.3%), Dizziness (62.2%), Skin rash (67.6%), sexual weakness associated with a spontaneous ejaculation, Nausea & vomiting (58.9%)  Sleeping troubles (69.4%), Nervousness (73.9%), Muscles aches (54.9%), Stomach cramps (60.4%), Headaches, Feeling cold and intense asthenia |  |  |  |
| 24 | Chikezie & Ebuenyi, (2019) [24] | Nigeria | A case reported at the emergency room | Case report | Case 1: Restlessness, Irrational and incoherent speech, destructive behaviour, insomnia, loss of appetite, visual and auditory hallucination, hyperactivity. Altered level of consciousness.  Case 2: Sudden collapse, convulsions and loss of consciousness (1400mg of Tramadol ingested), respiratory distress, incoherent speech, tonic-clinic seizure.  Case 3: Loss of consciousness, incomprehensive speech Restless  Case 4: Dizziness, Incoherent and irrational speech, altered level of consciousness, Restless  Case 5: Dizziness, excessive daytime sleepiness, unstable mood. |  |  |  |
| 25 | Chinweuba et al., (2023) [25] | Nigeria | Adolescents | Cross-sectional study | Not reported | High school students (n=55.76%)  12.1% |  |  |
| 26 | Chioma OO et al., (2022) [26] | Nigeria | Adolescents in summer camp (10 - 19 years) | Cross-sectional study | Not reported | Adolescents who take substances (n=28)  10.7% |  |  |
| 27 | Dada et al. (2021) [27] | Nigeria | Truck drivers | Cross-sectional study | Not reported | Truck drivers (n=306)  35.1% |  |  |
| 28 | Danso & Anto (2021) [28] | Ghana | Commercial drivers & assistants | Mixed method: cross-sectional study and focus group (qualitative research) |  | Commercial drivers & assistants (n=458)  24.9% | Among tramadol users, lever of risk of dependence (n=114)  49.1% |  |
| 29 | Diallo et al. (2021) [29] | Mali | Clients of pharmacies/Dispensaries | Cross-sectional study | Not reported | According to pharmacy patients (n=150)  11.3% | According to the patient at point G University (n=12)  24% | According to Pharmacists (n=30)  10.8% |
| 30 | Dumbili (2020) [30] | Nigeria | Youth adults (23-29 years) | Qualitative research | Loss of appetite, lose much weight, Appear unwell due to eating disorder, Loss of control, Seizure and convulsion, death |  |  |  |
| 31 | Ehwarieme & Emina, (2022) [31] | Nigeria | Patients admitted into Neuro-Psychiatric Hospital | Descriptive retrospective survey | Not reported | Patients admitted into Neuro-Psychiatric Hospital (n=1905)  0.6% |  |  |
| 32 | El Galad Abd Eldayed & Abd Elaziz (2018) [32] | Egypt | Drivers | Cross-sectional study | Not reported | Drivers (n=200)  11.5% |  |  |
| 33 | El Wasify et al. (2018) [33] | Egypt | Patients with tramadol dependence | Cross-sectional study | 69% have insomnia and sleep disturbances (n=400) |  |  |  |
| 34 | Elbeh et al. (2021) [34] | Egypt | Tramadol Drug Addicts | Cross-sectional study | Tramadol addiction is associated with a reduced volume of the brain's white matter, gray matter and cortical thickness. All frontal lobe regions have shown significant volume reduction in the tramadol group.  Tramadol addiction and its duration are associated with a risky decision-making style and higher impulsivity than non-addicts. |  |  |  |
| 35 | El-Gohari et al. (2022) [35] | Egypt | Inpatients and outpatients with (axis-I) psychiatric disorders | Cross-sectional study | Not reported | Patients admitted at Psychiatric hospital (n=168)  48.8% |  |  |
| 36 | Elhammady et al. (2014) [36] | Egypt | Inpatients with opioid dependency | Cross-sectional study | High rates of risky sexual behaviours were observed, particularly in tramadol users. They reported high rates of sexual activity over the previous three months, commercial sex work, a large number of casual sex partners, and lifetime hepatitis C. | Patients with opioid dependency (n=48)  77.1% |  |  |
| 37 | El-Safty et al. (2018) [37] | Egypt | OutPatient attending for treatment of Drug addiction | Cross-sectional study | Tramadol addiction causes reabsorption impairment of the nephron function regarding urinary calcium. |  |  |  |
| 38 | EL-Zoghby et al. (2017) [38] | Egypt | Adults attending the Family medicine center | Cross-sectional study | Not reported | Adults attending the family medicine center (n=243)  9.87% |  |  |
| 39 | Fasoro et al. (2020) [39] | Nigeria | Commercial motorcyclist | Cross-sectional study | Not reported | Commercial motorcyclist (n=89)  29.2% |  |  |
| 40 | Fuseini et al. (2019) [40] | Ghana | Patients with tramadol dependence | Qualitative research | Vomiting, Loss of Appetite, Seizure, Emotional aloofness, Irritability |  |  |  |
| 41 | Gallois et al. (2021) [41] | Cameroon | Baka communities/Cameroon's indigenous communities | Mix methods: observation and interviews (individual and group) | Sleepy, Itching, scraping the body, convulsion, death  Losing weight |  |  |  |
| 42 | Halawa, (2013) [42] | Egypt | Patients presented to the Poison Control Center (for Tramadol) | Cross-sectional study | Intoxication: Nausea, vomiting, coma, constricted Pupil, seizures, respiratory failure, death, pulse (tachycardia, bradycardia), Blood pressure (Shock, hypotension, hypertension), Respiratory rate (bradypnea, apnea, tachypnea), temperature (hypothermia, hyperthermia), Skin (Cyanosis, sweating), Pupil (constricted, dilated). Neurological system (coma, seizure, disequilibrium, agitation, hallucination), Respiratory system (respiratory distress, pulmonary edema), Cardiovascular system (Tachycardia, Myocardial ischemia, ventricular arrhythmia), GIT System (Nausea & vomiting) | Patients presented to the Poison Control Center (n=1581)  56.6% were addicted.  26.6% were suicidal attempt |  |  |
| 43 | Hassaan et al. (2021) [43] | Egypt | Patients with Tramadol-dependent | Prospective study with control | Tramadol dependence negatively affects cognitive performance, which improves with extended abstinence. |  |  |  |
| 44 | Ibrahim et al. (2017) [44] | Nigeria | Patients attending the addiction clinic of Federal Neuropsychiatric Hospital | Cross-sectional study | Not reported | Patients attending the addiction clinic (n=237)  54.4% and 78% of them met the IDC-10 diagnostic criteria for Tramadol dependence |  |  |
| 45 | Ibrahim et al. (2018) [45] | Nigeria | Female patient at the Federal Neuropsychiatric Hospital | Cross-sectional study | Not reported | Female with psychoactive substances dependence (n=253)  16.2% |  |  |
| 46 | Idowu A et al. (2018) [46] | Nigeria | Secondary Schools Students | Cross-sectional study | Not reported | The overall history of substance abuse (n=31)  12.9% |  |  |
| 47 | Ikenna et al. (2022) [47] | Nigeria | Students of the College of Medicine | Cross-sectional study | Not reported | Drug abuser Students (n=21)  4.4% |  |  |
| 48 | Iorfa et al. (2019) [48] | Nigeria | Tramadol abusers | Qualitative research | Value of life significantly negatively correlated with tramadol use but positively correlated with the dimensions of moral identity.  Tramadol abuse was significantly associated with value for life.  Moral identity moderated the relationship between tramadol use and value for life. | Not reported |  |  |
| 49 | Isabu & Iwuagwu, (2021) [49] | Nigeria | Youth (15-35 years) | Mixte method (Descriptive cross-sectional and Interviews and Focus Group) | Not reported | Semi-Urban youth (n=100)  21.7% |  |  |
| 50 | Johnson et al., (2017) [50] | Nigeria | Undergraduate Students | Cross-sectional study | Not reported | Undergraduate Students substance users (n=89)  74.2% |  |  |
| 51 | Kabbash et al. (2022) [51] | Egypt | Students of all faculties | Cross-sectional study | Not reported | Students (n=7445)  3% |  |  |
| 52 | Kabbash et al. (2022) [52] | Egypt | Students from all faculties | Cross-sectional study | Not reported | Students from all faculties (n=2552)  1% |  |  |
| 53 | Khafagy et al. (2021) [53] | Egypt | Students | Cross-sectional study | Not reported | Students substance users (n=74)  18.9% |  |  |
| 54 | Maiga et al. (2012) [54] | Niger | Teenagers and Young Adults living on the streets | Cross-sectional study | Not reported | Teenagers and Young Adults living on the streets (n=61)  77.04% |  |  |
| 55 | Maiga et al. (2013) [55] | Niger | Local councillors, administrators & street vendors | Qualitative research | Seizures, psychiatric disorders, deaths, brawls, kidnappings and rapes, murders, traffic accidents |  |  |  |
| 56 | Maigida & Hassan, (2019) [56] | Nigeria | Internally Displaced | Cross-sectional study | Not reported | Internally Displaced Substance use disorder (n=38)  10.5% |  |  |
| 57 | Makloph & Masoud, (2021) [57] | Egypt | Coffee Shop Workers | Cross-sectional study | Not reported | Coffee Shop Workers (n=120)  13.1% | Drug abusers (n=40)  40% |  |
| 58 | Makput et al. (2017) [58] | Nigeria | Female patients admitted to the Centre for Addiction Treatment | Retrospective study (Cross-sectional study) | Not reported | Female substance abusers (n=15)  42.2% |  |  |
| 59 | Mansour H et al., (2020) [59] | Egypt | Patients admitted with acute myocardial infarction | Cross-sectional study | Tramadol use was an independent predictor for left anterior descending artery (LAD) | Patients admitted with acute myocardial infarction (n=106)  29% |  |  |
| 60 | Meray et al. (2016) [60] | Egypt | Students | Cross-sectional study | Not reported | Students in Sohag University (n=500)  1.8% |  |  |
| 61 | Metuge et al. (2022) [61] | Cameroon | Students in tertiary schools | Cross-sectional study | Not reported | Students in tertiary schools (n=625)  7.5% Lifetime |  |  |
| 62 | Moussadak et al., (2021) [62] | Morocco | Psychotropic Medicines consumers | Cross-sectional study | Not reported | Psychotropic Medicines consumers (n=500)  47.80% |  |  |
| 63 | Naguib YM et al. (2021) [63] | Egypt | University Students | Cross-sectional study | Not reported | Substance-abusing Students (n=116)  31.1% |  |  |
| 64 | Negm & Fouad (2014) [64] | Egypt | Secondary School Students (13-18 years old) | Cross-sectional study | Not reported | Secondary School Students substance abuser (n=17)  83.3% |  |  |
| 65 | Ngwa (2022) [65] | Nigeria & Cameroon | Teachers, Addicts-Students, Illegal vendors, Medical Practitioners, Rehab Experts | Qualitative research | Sleepiness, Unconsciousness, Coma, Seizures, Respiratory Problems, Low Blood pressure, Weak muscles.  Irritability, Anger, Anxiety, Emotional aloofness.  Aggressive behaviours, Violence among peers, Street Violence, Sexual Assault/Rape, Theft, suicide, and Death. |  |  |  |
| 66 | Nwala (2021) [66] | Nigeria | Adolescent Students (Rural & Urban) | Cross-sectional study | Not reported | Adolescent Students abusers (n=154)  6.3% |  |  |
| 67 | Offie et al. (2022) [67] | Nigeria | Young People among 10 - 24 years | Cross-sectional study | Not reported | Young People among 10 - 24 years (n=290)  6.6% (current use) | Young People among 10 - 24 years (n=315)  1.9% (lifetime use) |  |
| 68 | Olanrewaju JA et al., (2022) [68] | Nigeria | Undergraduate Students | Cross-sectional study | Not reported | Undergraduate Students (n=400)  35.0% |  |  |
| 69 | Omilani et al. (2021) [69] | Nigeria | Patients attending a psychiatric clinic | Cross-sectional study | Not identified | Patients attending psychiatric clinic (n=127)  46.9% |  |  |
| 70 | Onu et al. (2021) [70] | Nigeria | Students of a federal university in the Southeast region of Nigeria | Cross-sectional study | Adverse childhood experiences (ACE) were positively associated with tramadol use. Sociosexual behaviours and desires mediated the relationship between ACEs and tramadol use. | Not reported |  |  |
| 71 | Peprah et al., (2020) [71] | Ghana | Commercial & assistants vehicle drivers | Qualitative research | 1) Physical: seizure, vomiting, anorexia, loss of appetite, hallucination, severe Nausea, agitation & confusion, drowsiness, dry mouth, headache, loss of strength, muscle aches, joint pain, severe redness, swelling, itching of the skin, sweating, swelling of the hands, ankles and feet, legs, trembling and shaking of the hand or feet, week or absence of a pulse in the legs.  2) Psychological: irritability, anger, overactive reflexes, loss of consciousness, discouragement, general discomfort, sadness and loss of interest or pleasure.  3) Social effects: Social stigma, lack of respect |  |  |  |
| 72 | Saapiire et al., (2021) [72] | Ghana | The active population between the ages 15-55 years | Cohort study | Not reported | Active population within the age 15-55 years (420)  36.2% with 77.6% of misuse |  |  |
| 73 | Sarfo, (2020) [73] | Ghana | Senior high school students | Cross-sectional study/Thesis | Not reported | Senior high school students  7.5% |  |  |
| 74 | Shalaby AS et al. (2015) [74] | Egypt | Tramadol users | Cohort study | Tramadol increases the severity of nicotine dependence. The relation seems bi-directional, so increased cigarette smoking also increases tramadol intake. | Not reported |  |  |
| 75 | Shamloul RM et al., (2020) [75] | Egypt | Patients with seizures | Cross-sectional study | Tramadol-induced seizures represented 7% of all patients (103 out of 1480) and 12% of males. Generalized tonic conic seizures were the most common type of tramadol-induced seizures (86%), and focal seizures occurred only in 14% of patients. There was no history of status epilepticus in any patient. | Not reported |  |  |
| 76 | Sy et al. (2020) [76] | Senegal | Patient with addiction to Tramadol in sickle cell | Case report | Dependence syndrome: Insomnia  Withdrawal symptoms: severe pain, craving, a feeling of fatigue, Palpitations and tremors |  |  |  |
| 77 | Taha et al. (2019) [77] | Egypt | Patients presented with a psychotic episode | Cohort study | Not reported | Patients presented with psychotic episodes (n=81)  4.9% | Patients with bipolar illness (n=38)  2.6% | Patients with  Schizo-Effective  Disorders (n = 27)  7.4% |
| 78 | Toufique (2022) [78] | Ghana | Tricycle drivers | Cross-sectional study/Dissertation/Thesis | Aggressiveness, anxiety and depression, sleepiness/Dizziness, Inattentiveness/Inability to focus, Respiratory failure, dependence to Drugs) |  |  |  |
| 79 | Udo (2022) [79] | Nigeria | Senior Secondary Student | Cross-sectional study | The finding reveals that a negative relationship exists between Tramadol abuse on students disruptive behaviour among Senior Secondary students in the Uyo metropolis of Akwa-Ibom state r = -0.228, p = 0.033. |  |  |  |
| 80 | Umukoro et al. (2021) [80] | Nigeria | Students from Secondary School | Cross-sectional study | Not reported | Students from Secondary School (n=315)  10.79% |  |  |
| 81 | Uwaibi et al. (2022) [81] | Nigeria | Young People (19-35 years old) communities | Cross-sectional study | Not reported | Knowledge of substance abuse (n=412)  11.2% | Respondents who take drugs (n=29)  41.4% |  |
| 82 | Yakubu-Wokili et al. (2022) [82] | Nigeria | Commercial motorcycle operator | Cross-sectional study | Not reported | Commercial motorcycles (n=372)  76% |  |  |
| 83 | Yunusa (2017) [83] | Nigeria | Commercial Bus Drivers | Cross-sectional study | Not reported | Commercial Bus Drivers (n=196)  19.4% |  |  |

*^1^Pop 1: First Studies Population 1; ^2^Pop 2 :Second studied Population; ^3^Pop3 : Third studied Population*

**References**

1. Abbas RA, Hammam RA, El-Gohary SS, Sabik LM, Hunter MS. Screening for common mental disorders and substance abuse among temporary hired cleaners in Egyptian Governmental Hospitals, Zagazig City, Sharqia Governorate. Int J Occup Env Med. 2013;4: 13–26.

2. Abdel Kareem R, Ali D. Prevalence Of Drug Abuse Among Drivers In Minia Governorate. Egypt J Forensic Sci Appl Toxicol. 2018;18: 115–133. doi:10.21608/ejfsat.2018.3876.1013

3. Abdel Moneim A, Abdullah E, Rashwan M. Study of Drug Abuse among Trauma Patients in Sohag University Hospitals. Sohag Med J. 2017;21: 219–223. doi:10.21608/smj.2017.53477

4. Abdelfattah H, Abdelaal E, Sobhy D. Assessment of Non- Medical Use of Tramadol among University Students. Menoufia Nurs J. 2019;4: 93–102. doi:10.21608/menj.2019.156133

5. Abdelhamid BM, Mostafa Gomaa H, Abdelgaleel AG, Hussein W, Mohamed Ali H. Prevalence of drug abusers in patients undergoing elective surgeries at the Cairo University Teaching Hospital; Prospective cohort study. Egypt J Anaesth. 2022;38: 131–138. doi:10.1080/11101849.2022.2036526

6. Abd-Elkader MR, Kabbash IA, El-Sallamy RM, El-Sawy H, Gad EA. Tramadol abuse among workers in an industrial city in mid-Nile Delta region, Egypt. Env Sci Pollut Res Int. 2020;27: 37549–37556. doi:10.1007/s11356-020-08040-8

7. Amin M, Abd-Elwahab M. Sexual risk among substance users and its relation to personality profile. Egypt J Psychiatry. 2012;33: 135. doi:10.7123/01.EJP.0000415088.97056.8a

8. AbdelWahab MA, Abou El Magd SF, Grella CE, Enaba DA, Abdel Maqsoud R. An examination of motives for tramadol and heroin use in an Egyptian sample. J Addict Dis. 2018;37: 123–134. doi:10.1080/10550887.2019.1623650

9. Agberotimi S, Asagba R, Oduaran C, Folorunso S. Non-medical use of pharmaceutical drugs at workplace among skilled workers. J Subst Use. 2020;25: 658–662. doi:10.1080/14659891.2020.1760375

10. Amankwaa P. The Use Of Illicit Drugs And Their Effects On Academic Performance Among Senior High School Students In Sunyani West District. 2019.

11. Argungu ZM, Sa’idu A, Sanda LU. Effect of Tramadol on Sexual Life Quality of Tramadol Users in Nigeria. 2021.

12. Azab SMS, Tawfik H, Hayes BD. Intoxication related to substances use in patients presenting to Ain Shams University Poisoning Treatment Center, Cairo, Egypt (2015–2019). Drug Alcohol Rev. 2022;41: 1109–1118. doi:10.1111/dar.13443

13. Bassiony M, Seleem D. Drug-related problems among polysubstance and monosubstance users: a cross-sectional study. J Subst Use. 2020;25: 392–397. doi:10.1080/14659891.2020.1720326

14. Bassiony M, Mahdy RS, Haggag N, Salah Eldeen G. Current attention-deficit/hyperactivity disorder and nonmedical prescription opioid use attributed to tramadol among male adolescents without conduct disorder in Egypt. Am J Drug Alcohol Abuse. 2021;47: 623–629.

15. Bassiony M, Salah El-Deen GM, Yousef U, Raya Y, Abdel-Ghani MM, El-Gohari H, et al. Adolescent tramadol use and abuse in Egypt. Am J Drug Alcohol Abuse. 2015;41: 206–211. doi:10.3109/00952990.2015.1014959

16. Bassiony M, Youssif U, Hussein R, Saeed M. Psychiatric Comorbidity Among Egyptian Patients With Opioid Use Disorders Attributed to Tramadol. J Addict Med. 2016;10: 262–8. doi:10.1097/ADM.0000000000000231

17. Bassiony M, El-Deen S, Ameen N, Mahdy R. Prevalence, correlates, and consequences of attention-deficit/hyperactivity disorder in a clinical sample of adults with tramadol use in Egypt. Am J Addict. 2022;31: 31–36. doi:10.1111/ajad.13231

18. Bassiony M, Abdelghani M, Salah El-Deen GM, Hassan MS, El-Gohari H, Youssef UM. Opioid Use Disorders Attributed to Tramadol Among Egyptian University Students. J Addict Med. 2018;12: 150–155. doi:10.1097/ADM.0000000000000380

19. Bassiony M, Salah El-Deen G, Abdelghani M, Youssef UM, Hassan MS, El-Gohari H. Addressing prevalence and correlates among a sample of Egyptian university students who suffer from substance use disorders. Addict Disord Their Treat. 2018;17: 114–123.

20. Bio-Sya A, Damien GB, Kpatchavi AC, Allabi AC. Prevalence, associated factors and level of dependence of substance use among urban secondary school students, Benin. Basic Clin Pharmacol Toxicol. 2022;131: 205–213. doi:10.1111/bcpt.13764

21. Bio-Sya A, Gandjo HJ, Klikpo ETE, Kouglenou O, Agbanlinsou AC, Damien GB, et al. Nonmedical use of tramadol among secondary school students in Benin, Africa. Am J Drug Alcohol Abuse. 2021;47: 746–752. doi:10.1080/00952990.2021.1955897

22. Burbwa SN, Kimbi DN. Drug Abuse among “Okada” Riders Business Youths in Katsina-Ala Local Government Area of Benue State, Nigeria. Niger J Health Promot. 2019;12.

23. Carmel AG, Diallo A, Essotolom B, Gbeasor FA, Lawson-evi P, Ekouevi DK, et al. Misuse of tramadol among motorcycle taxi drivers of Lome (Togo). 2019;7: 5.

24. Chikezie U, Ebuenyi ID. Tramadol misuse in the Niger Delta; A review of cases presenting within a year. J Subst Use. 2019;24: 487–491. doi:10.1080/14659891.2019.1604842

25. Chinweuba DC, Ifeagwazi CM, Chinweuba AU, Chukwuorji JC. Does self-concealment and self-compassion differentially influence substance use for male versus female adolescents? J Subst Use. 2023;28: 117–122. doi:10.1080/14659891.2021.2018726

26. Chioma OO, Bridget IU, Ifeyinwa CN, Fidelia OE. Knowledge and indulgence in substance abuse among adolescents in Anambra state, South-East Nigeria. Afr Health Sci. 2022;22: 227–33.

27. Dada MU, Adeoti AO, Elebiyo T, Kumolalo BF`, Ajiboye AS. Pattern of Psychoactive Substances Use among Long-Distance Truck Drivers in Nigeria. Asian J Med Health. 2021; 18–25. doi:10.9734/ajmah/2021/v19i130293

28. Danso M, Anto F. Factors Associated with Tramadol Abuse: A Cross-Sectional Study Among Commercial Drivers and Assistants in the Accra Metropolitan Area of Ghana. Drugs - Real World Outcomes. 2021;8: 337–347. doi:10.1007/s40801-021-00247-6

29. Diallo T, Diara A, Coulibaly SK, Konaté Y, Hami H, Soulaymani A, et al. Misuse of drugs in the District of Bamako, Mali. Bourekkadi S, Hami H, Mokhtari A, Slimani K, Soulaymani A, editors. E3S Web Conf. 2021;319: 02019. doi:10.1051/e3sconf/202131902019

30. Dumbili EW. Drug-related harms among young adults in Nigeria: Implications for intervention. J Hum Behav Soc Environ. 2020;30: 1013–1029. doi:10.1080/10911359.2020.1790462

31. Ehwarieme T, Emina A. Prevalence of Psychoactive Substance use and its Contributing Factors among Patients Admitted into the Federal Neuro-Psychiatric Hospital, Benin City: A Retrospective Study. J Med Basic Sci Res. 2022;3: 41–53.

32. El Galad G, Abd Eldayed A, Abd Elaziz M, El Said S. Detection of Drugs of Abuse among Drivers in Fayoum City/ Egypt. Ain Shams J Forensic Med Clin Toxicol. 2018;31: 94–99. doi:10.21608/ajfm.2018.15882

33. El Wasify M, Fawzy M, Barakat D, Youssef U, El Wasify M, Saleh A, et al. The Sociodemographic and Clinical Characteristics of Tramadol Dependence among Egyptians and Their Relationship to the Associated Insomnia. Addict Disord Their Treat. 2018;17: 98–106. doi:10.1097/ADT.0000000000000129

34. Elbeh KA, Elserogy Y, Seifeldein GS, Mostafa S, Yousef HA, Fawzy M. Impulsivity and Decision-making Style Among Tramadol Drug Addicts and its Relation to Frontal Lobe Volume. Addict Disord Their Treat. 2021;20: 314–325.

35. El-Gohari HM, Fouad E, Abdelghani M. Opioid use disorders related to tramadol among an Egyptian sample of male psychiatric patients: prevalence and sociodemographic and clinical correlates. J Subst Use. 2022;27: 50–55. doi:10.1080/14659891.2021.1892220

36. Elhammady M, Mobasher M, Moselhy HF. Pattern of risky sexual behaviors in opioid-dependent egyptian adults. Addict Disord Their Treat. 2014;13: 68–74.

37. El-Safty I, Eltamany E, Shouman A, El-Gamel O, Nada A, Ali W. Effect of tramadol addiction alone and its co-abuse with cannabis on urinary excretion of Copper, Zinc, and Calcium among Egyptian addicts. Afr Health Sci. 2018;18: 767. doi:10.4314/ahs.v18i3.35

38. EL-Zoghby SM, Mansour NM, Eldahshan NA, Fawzy MS, Hagras AM. Pattern of illicit drug use among adults attending family Medicine Center Of Fanarah village in Ismailia City, Egypt. Egypt J Forensic Sci Appl Toxicol. 2017;17: 1–20. doi:10.21608/ejfsat.2017.45678

39. Fasoro O, Olusuyi A, Dada A, Adewumi M, Oluwatuyi V. Assessing Substance Abuse among Commercial Mo-torcyclists in Ijero Township, Ekiti State, Southwestern Nigeria: It’s Implication to Public Health. J Addict Ther JATP-126 DOI. 2020;10: 2577–1507.

40. Fuseini AG, Afizu A, Yakubu YH, Nachinab G. Facilitators to the continuous abuse of tramadol among the youth: A qualitative study in Northern Ghana. Nurs Open. 2019;6: 1388–1398. doi:10.1002/nop2.353

41. Gallois S, Van Andel TR, Pranskaityté G. Alcohol, drugs and sexual abuse in Cameroon’s rainforest. Soc Sci Med. 2021;277: 113929. doi:10.1016/j.socscimed.2021.113929

42. Halawa H. Clinical study of acute tramadol poisoning in the poison control center, Ain Shams University in 2012. Ain Shams J Forensic Med Clin Toxicol. 2013;21: 20–26.

43. Hassaan SH, Khalifa H, Darwish AM. Effects of extended abstinence on cognitive functions in tramadol‐dependent patients: A cohort study. Neuropsychopharmacol Rep. 2021;41: 371–378. doi:10.1002/npr2.12188

44. Ibrahim A, Yerima M, Pindar S, Onyencho V, Ahmed H, Machina B, et al. Tramadol abuse among patients attending an addiction clinic in North-Eastern Nigeria: outcome of a four year retrospective study. Adv Psychol Neurosci. 2017;2: 31–37.

45. Ibrahim AW, Pindar SK, Shettima FB, Mshelia AA, Amodu MO, Machina BK, et al. Psychoactive Substance Use Disorders Among Females in Northern Nigeria: Findings Of A Five-Year Descriptive Survey at The Federal Neuropsychiatric Hospital, Maiduguri. African Journal of Drug & Alcohol Studies. 2018;17.

46. Idowu A, Aremu AO, Olumide A, Ogunlaja AO. Substance abuse among students in selected secondary schools of an urban community of Oyo-state, South West Nigeria: implication for policy action. Afr Health Sci. 2018;18: 776. doi:10.4314/ahs.v18i3.36

47. Ikenna GE, Brian OO, Nneoma NO. Identification of substance abuse among medical students in a Nigerian University. IJMS. 2022;6: 21.

48. Iorfa SK, Ifeagwazi CM, Effiong JE, Essien NF. Tramadol abuse and value for life among young persons: Moderating effects of moral identity. Afr J Drug Alcohol Stud. 2019;18: 109–120.

49. Isabu AC, Iwuagwu TE. Social Environmental Factors Associated with the Recent Surge in Psychoactive Substance Abuse among Youths in a Selected Semi-Urban Community in South-South Nigeria. Niger J Health Promot. 2021;14.

50. Johnson O, Akpanekpo E, Okonna E, Adeboye S, Udoh A. The prevalence and factors affecting psychoactive substance use among undergraduate students in University of Uyo, Nigeria. J Community Med Prim Health Care. 2017;29: 11–22.

51. Kabbash I, Zidan O, Saied S. Substance abuse among university students in Egypt: prevalence and correlates. East Mediterr Health J. 2022;28: 31–40.

52. Kabbash I, Zidan O, Younis E. Drug use among students of Tanta University: prevalence and correlates. J Subst Use. 2022; 1–7.

53. Khafagy M, Gomaa Z, Elwasify M. Substance use patterns among university students in Egypt. Middle East Curr Psychiatry. 2021;28: 1–9.

54. Maiga D, Seyni H, Moussa AO, Sidikou A. Mesusage du tramadol par les adolescents et jeunes adultes en situation de rue. Pan Afr Med J. 2012;13: 55.

55. Maiga D, Seyni H, Sidikou A. Social representations of consumption of tramadol in Niger, perceptions and knowledge of communities: issues for action. Afr J Drug Alcohol Stud. 2013;12: 53–61.

56. Maigida K, Hassan A. Prevalence and pattern of substance use among internally displaced persons in north-central Nigeria. Drugs Niger Popul. 2019;62: 49.

57. Makloph M, Masoud M. Drug Abuse Prevalence and Effects in Coffee Shop Workers: A Cross Sectional Study. Indian J Forensic Med Toxicol. 2021;15: 1713–1721.

58. Makput D, Okonoda K, Maigari Y, Piwuna C, Nantok D, Davou F, et al. Psychoactive substance dependence in females. A three year review of patients on admission at an addiction treatment centre in North Central Nigeria. Niger J Med. 2017;26: 360. doi:10.4103/1115-2613.302792

59. Mansour H, Rayan M, Shnoda M, Kamal D. Cannabis and tramadol addiction: Do they imply additive risk for acute myocardial infarction in adults younger than 45 years? Anatol J Cardiol. 2020;24: 316–325. doi:10.14744/AnatolJCardiol.2020.67206

60. Meray M, Ahmed F, Rania A. Study of drug abuse among university students in Sohag, Egypt. J Forensic Toxicol Medicolegal Anal. 2016;1: 19–23.

61. Metuge CE, Dzudie A, Ebasone PV, Assob JCN, Ngowe MN, Njang E, et al. Prevalence and factors associated with substance use among students in tertiary institutions in Buea, Cameroon. Pan Afr Med J. 2022;41. doi:10.11604/pamj.2022.41.103.29272

62. Moussadak A, Farhane H, Benaji B, Bouzoubaa H, Houti I, El Omari F, et al. Toxicovigilance: the misuse of psychotropic drugs in Morocco. Results of a survey conducted in Casablanca. Bourekkadi S, Hami H, Mokhtari A, Slimani K, Soulaymani A, editors. E3S Web Conf. 2021;319: 01056. doi:10.1051/e3sconf/202131901056

63. Naguib YM, Sherif HA, Elbalshy AT, Edrees EA, Sabry AE, Sharif AF, et al. Prevalence and associated risk factors of cannabinoid abuse among Egyptian university students: a cross-sectional study. Env Sci Pollut Res Int. 2021;28: 68706–68716. doi:10.1007/s11356-021-15412-1

64. Negm MG, Fouad AA. Prevalence of substance abuse among adolescent school students in Zagazig. Egypt J Psychiatry. 2014;35: 161.

65. Ngwa NR. Drug Abuse and its Implication on Regional Security in West and Central Africa: Cases Studies of Nigeria and Cameroon. Int J Polit Secur. 2022 [cited 3 May 2023]. doi:10.53451/ijps.1024349

66. Nwala G, Ibeneme C, Ojinnaka N, Ugolee J. Changing trend of psychoactive drug abuse among adolescent students in South Eastern Nigeria. Journal of Drug Abuse. 2021;7: 30. doi:10.36648/2471-853X.7.4.30

67. Offie DC, Oluwatobi A, Ekanem EE, Dike PN, Oguh DN, Adijat OS. Patterns of substance use among young people in Ado Ekiti, Southwest, Nigeria. Texila Int J Public Health. 2022;10. doi:10.21522/TIJPH.2013.10.02.Art024

68. Olanrewaju JA, Hamzat EO, Enya JI, Udekwu MO, Osuoya Q, Bamidele R, et al. An assessment of drug and substance abuse prevalence: a cross-sectional study among undergraduates in selected southwestern universities in Nigeria. J Int Med Res. 2022;50: 3000605221130039. doi:10.1177/03000605221130039

69. Omilani A, Moses A, Iorbo M. Prevalence Of Drug Abuse Among Patients Attending Psychatric Clinic At Federal Medical Centre, Makurdi. Sci Prepr. 2021.

70. Onu DU, Ifeagwazi CM, Orjiakor CT, Iorfa SK. Adverse childhood experiences and tramadol use in Nigeria: the mediating role of sociosexuality in a predominantly male student sample. J Subst Use. 2021;26: 427–433. doi:10.1080/14659891.2020.1846805

71. Peprah P, Agyemang-Duah W, Appiah-Brempong E, Akwasi AG, Morgan AK. “with tramadol, i ride like a Jaguar”: A qualitative study of motivations for non-medical purpose tramadol use among commercial vehicle operators in Kumasi, Ghana. Subst Abuse Treat Prev Policy. 2020;15. doi:10.1186/s13011-020-00292-4

72. Saapiire F, Namillah G, Tanye V, Abubakari A. The Insurgence of Tramadol Abuse among the Most Active Population in Jirapa Municipality: A Study to Assess the Magnitude of the Abuse and Its Contributory Factors. Grutsch J, editor. Psychiatry J. 2021;2021: 1–10. doi:10.1155/2021/3026983

73. Sarfo G. Prevalence of cigarette, alcohol, marijuana and tramadol use among senior high school students in the Bekwai Municipality, Ghana. Master of Philosophy degree in Guidance and Counselling, University of Cape Coast. 2020. Available: https://ir.ucc.edu.gh/xmlui/handle/123456789/5016

74. Shalaby AS, El-Hady Sweilum OA, Ads MK. Does Tramadol Increase the Severity of Nicotine Dependence? A Study in an Egyptian Sample. J Psychoact Drugs. 2015;47: 197–202. doi:10.1080/02791072.2015.1050534

75. Shamloul RM, Elfayomy NM, Ali EI, Elmansy AMM, Farrag MA. Tramadol-associated seizures in Egypt: Epidemiological, clinical, and radiological study. Neurotoxicology. 2020;79: 122–126. doi:10.1016/j.neuro.2020.05.002

76. Sy A, Tine JAD, Benmansour S, Mamadou SO, Binta B, Aissatou D, et al. Sickle Cell Patient and Addiction to Tramadol: Case Management in Senegal. Health (N Y). 2020;12: 99–105.

77. Taha M, Taalab YM, Abo-Elez WF, Eldakroory SA. Cannabis and tramadol are prevalent among the first episode drug-induced psychosis in the Egyptian Population: Single center experience. Y Rep. 2019;2: 16.

78. Toufique S. Factors influencing the abuse of tramadol among tricycle (Yeloyelo) drivers in the Tamale metropolis. Desertation/Thesis submitted, University for development studies. 2022. Available: http://www.udsspace.uds.edu.gh/bitstream/123456789/3962/1/FACTORS%20INFLUENCING%20THE%20ABUSE%20OF%20TRAMADOL%20AMONG%20TRICYCLE.pdf

79. Udo M-AA. Relationship between drug abuse and students disruptive behaviour among secondary school students in Uyo Metropolis Of Akwa-Ibom State, Nigeria. Nigerian Journal of Arts and Humanities. 2022;2.

80. Umukoro EK, Eduviere AT, Ahama EE, Moke EG, Edje KE, Omorodion LI, et al. Substance Abuse: Awareness and Attitude among Secondary School Students in Sapele, Nigeria. J Appl Sci Environ Manag. 2021;25: 347–351. doi:10.4314/jasem.v25i3.7

81. Uwaibi NE, Omozuwa ES, Agbonrofo-Eboigbe GE. Prevalence, Sociodemograhic Characteristics and Substance Abuse among Young Persons in Edo State, Nigeria. J Appl Sci Environ Manag. 2022;26: 361–367. doi:10.4314/jasem.v26i2.26

82. Yakubu-Wokili H, Owoeye AS, Jatau SU, Oni BG, Kayode SI. Assessment of Psychoactive Substance Abuse Among Commercial Motorcycle Operators in Minna, Nigeria. 2022.

83. Yunusa U, Bello UL, Idris M, Haddad MM, Adamu D. Determinants of Substance Abuse among Commercial Bus Drivers in Kano Metropolis, Kano State, Nigeria. Am J Nurs Sci. 2017;6: 125. doi:10.11648/j.ajns.20170602.16
