# Supplementary material for: Prevalence and health consequences of nonmedical use of tramadol in Africa: A systematic scoping review
Source: PLOS Glob Public Health. 2024 Jan 18;4(1):e0002784. doi: 10.1371/journal.pgph.0002784 (PMC10796000; doi:10.1371/journal.pgph.0002784)
Supplement: Attachment — Submitted filename: Response to reviewers.docx [file pgph.0002784.s005.docx]

PGPH-D-23-01629

Prevalence and health consequences of nonmedical use of Tramadol in Africa: A systematic scoping review

PLOS Global Public Health

Reviewer #1: The manuscript is worth publishing and I request the manuscript to be accepted. The authors have made a good attempt in writing the manuscript and the misuse of tramadol in the African countries and it is worth publishing.

Reviewer #2: Review Comments on Manuscript PGPH-D-23-01629: Prevalence and Health Consequences of Non-medical Use of Tramadol in Africa: A systematic Scoping Review

This is a well-researched scoping review. I applaud the efforts of the authors to put together a “pooled” prevalence of non-medical use of tramadol in Africa which is a continent most hit by opioid crisis in contemporary times. However, I believe the review manuscript would be made richer if the authors consider the following comments:

Abstract
1. The abstract should cover in statistical terms, the prevalence of non-medical use of tramadol in Africa, highlighting the percentages for each non-medical use as reported in the studies reviewed.

RESPONSE: Thank you for your comment. The prevalences of non-medical use of tramadol found in the study have been added to the abstract as requested.

2. The prevalence (in percentages) of NMU of tramadol among young adults, professionals, patients and academics should be stated.
RESPONSE: Thank you for your comment. The prevalences of non-medical use of tramadol among young adults, professionals, patients, and academics, found in the study have been added to the abstract as requested.

Introduction
1. Paragraph 1: Mention those countries where tramadol is put under control (especially in Africa).

RESPONSE: Thank you for your comment. In the introduction, we have listed the countries where tramadol is regulated.

2. Paragraph 1: Define non-medical use of tramadol.

RESPONSE: Thank you for your comment. We have added the definition of the non-medical use of Tramadol to the Introduction as requested.

3. Paragraph 3: mention some common brand names of tramadol in African countries.

RESPONSE: Thank you for your comment. We have added the brand names of tramadol in African countries as requested.
4. Has there been any review study on tramadol in Africa or elsewhere? What aspects have been covered and what is the gap for this review study to fill?

RESPONSE: Thank you for your comment. We have included information about the existing review and the aspects covered, as well as the gap filled by our review.

Results
1. The section on “Prevalence of Non-medical Use of Tramadol” should be presented before the section on “Health Consequences of Non-medical Use of Tramadol”.

RESPONSE: Thank you for your comment. We have made the change as requested.

Discussion
1. Under “Comparative literature”, paragraph 2: first line: different reference style used here: Iwanicki et al. (2020).

RESPONSE: Thank you for your comment. We have made the change as requested.

2. Third paragraph under “comparative literature” looks better if presented in the “Introduction”.

RESPONSE: Thank you for the comment. We have moved the relevant paragraph back to the introduction.

Conclusion
1. The conclusion is too lengthy. It should cover only the salient points drawn from the findings of the review study. It shouldn’t look like another “discussion” of findings.

RESPONSE: Thank you for the comment. We shortened the conclusion to highlight only salient findings.
